# Neurophysiological and Structural–Mechanical Changes Associated with Dry Needling in Post-Stroke Spasticity: A Systematic Review

**DOI:** 10.3390/jcm15114246

**Published:** 2026-05-30

**Authors:** Bart Eeckhaut, Steven Truijen, Caroline Leroij, Juliette Dévillé, Lisa Jacobs, Wim Saeys

**Affiliations:** Department of Rehabilitation Sciences and Physiotherapy, Faculty of Medicine and Health Sciences, University of Antwerp, Universiteitsplein 1, 2610 Antwerpen, Belgium; steven.truijen@uantwerpen.be (S.T.); caroline.leroij@student.uantwerpen.be (C.L.); lisa.jacobs@student.uantwerpen.be (L.J.); wim.saeys@uantwerpen.be (W.S.)

**Keywords:** stroke, spasticity, dry needling, structural and mechanical properties, neurophysiological effects

## Abstract

**Background/Objectives:** In the past few years increasing attention has been given to the application of dry needling (DN) for spasticity in stroke survivors. Nevertheless, the underlying mechanisms of this technique have not yet been confirmed. The aim of this systematic review was to distinguish the effects of DN in post-stroke spasticity on both structural–mechanical muscle properties (SMMPs) and neurophysiological properties to address these mechanisms. **Methods:** A literature search was performed in Web of Science, PubMed, Scopus and Embase following PRISMA guidelines (PROSPERO ID: 1163064). Randomized controlled trials and case–control studies involving adults with post-stroke spasticity treated with DN were included. Outcomes were categorized as SMMPs (e.g., muscle architecture, passive stiffness, PROM) or neurophysiological measures (e.g., H-reflex, H/M ratio). Standardized effect sizes (Hedges’ g) were calculated when possible; however, heterogeneity in outcomes and incomplete variance reporting precluded meta-analysis. **Results:** Twelve studies met the inclusion criteria. Most of these studies assessed passive range of motion, reporting a significant increase following the intervention. Only two of the included studies examined structural characteristics, and five studies included neurophysiological outcomes. Correlations between mechanistic outcomes and clinical spasticity grading (MAS/MMAS) were weak. Emerging evidence suggests DN may additionally modulate local inflammatory mediators, indicating a potential neuroimmune contribution to its effects. **Conclusions:** DN appears to improve structural–mechanical muscle properties and produce moderate reductions in reflex excitability in individuals with post-stroke spasticity. Mechanical adaptations are more consistently demonstrated than neural changes, and neither domain is proportionally reflected in clinical spasticity scales. Evidence remains limited by small samples, methodological variability, and incomplete reporting. Further mechanistic research is needed to clarify how DN influences the complex pathophysiology of post-stroke spasticity.

## 1. Introduction

One of the major impairments observed in neurological populations is abnormal muscle tone regulation. Within the umbrella of ‘hypertonia’, post-stroke spasticity (PSS) remains one of the most prevalent tone-related disorders after stroke. To date, the management of post-stroke spasticity continues to rely mostly on the definition provided by James Waldo Lance [1]. Despite its foundational significance in both the research literature and clinical guidelines, this definition does not fully capture the multidimensional nature of PSS nor the complex pathophysiological mechanisms underlying increased resistance to passive movement [2,3,4].

The first manifestations of PSS reflect both adaptive and maladaptive neuroplastic processes following stroke, as these neurophysiological changes typically include a decreased stretch reflex threshold [5]. These neurophysiological alterations differ from the structural and mechanical muscular property (SMMP) changes observed in chronic post-stroke survivors. SMMP changes arise as consequences of long-term muscle impairments such as muscle weakness and altered movement patterns. They manifest as reduced muscle thickness, altered visco-elastic properties, and increased muscle stiffness in the affected limb, all of which are strongly associated with spasticity. For example, ultrasonographic studies have demonstrated structural and contractile changes in spastic muscles, including reduced muscle thickness and altered pennation angles [6]. Furthermore, Pennati et al. (2016) identified a clear correlation between total passive range of motion (PROM) and spastic wrist flexors [7]. Another relevant aspect of PSS is muscle stiffness, which can be objectively quantified as passive torque, which is significantly elevated in individuals with PSS compared to healthy controls [8,9]. Passive torque differs from reflex torque, using low velocities during assessment.

Since stroke often results in complex and multifaceted motor impairments, multiple assessment tools are required to adequately characterize post-stroke spasticity within the broader framework of upper motor neuron syndrome [10]. The Modified Ashworth Scale (MAS) remains the most used clinical measure due to its convenience and time-efficiency. However, the MAS does not differentiate whether increased resistance arises from neural mechanisms, such as altered stretch reflex excitability, or from structural–mechanical muscle alterations [11]. Given that resistance to passive movement reflects a combination of neural and non-neural components, interpretation of treatment effects based solely on MAS scores may not clarify which underlying mechanisms are being influenced. These considerations highlight the importance of carefully selecting outcome measures when evaluating interventions targeting PSS.

Dry needling (DN) has emerged as a potential intervention for post-stroke spasticity [12,13,14]. DN involves the insertion of a fine solid needle into muscle tissue without the administration of medication. In contrast to traditional acupuncture, which is historically rooted in meridian-based concepts of traditional Chinese medicine, DN is grounded in Western biomedical principles and primarily targets myofascial trigger points or dysfunctional motor endplates within muscle tissue [15]. Although both techniques use similar instruments, they differ in their theoretical framework and clinical rationale. The proposed mechanisms of DN are multilevel. At the peripheral level, DN may modulate endplate activity, influence local biochemical mediators, and alter afferent sensory input, particularly when a local twitch response is elicited [16]. These peripheral effects are thought to contribute to changes in muscle mechanical properties and sensorimotor feedback. At the spinal level, DN has been hypothesized to influence reflex excitability and sensorimotor processing [17,18]. However, direct evidence supporting specific neurophysiological mechanisms in post-stroke populations remains limited. In addition to mechanical and neurophysiological mechanisms, emerging evidence suggests that dry needling may also influence neuroinflammatory processes. Modulation of local inflammatory mediators and cytokine activity has been proposed as a potential pathway contributing to changes in muscle function and neural excitability. This perspective further supports the multifactorial nature of dry needling effects and warrants consideration when interpreting mechanistic outcomes [19,20]. Consequently, it remains unclear whether reported reductions in spasticity following DN primarily reflect the modulation of neural reflex pathways or alterations in structural–mechanical muscle properties.

The aim of this systematic review was to distinguish the effects of DN in post-stroke spasticity on both structural–mechanical muscle properties (SMMPs) and neurophysiological properties to address these mechanisms. A secondary objective was to examine the relationship between clinical changes in spasticity grading and objective measures of structural–mechanical or neurophysiological function. By distinguishing between neural and peripheral mechanisms, this review aims to clarify the mechanistic basis of dry needling in post-stroke spasticity management.

## 2. Methods

This systematic review was conducted in accordance with the Preferred Reporting Items for Systematic Reviews and Meta-Analyses (PRISMA) 2020 statement, the completed PRISMA checklist is provided in the Supplementary Materials. The study was also prospectively registered in the PROSPERO database (registration number ID: 1163064) [21]. The review was completed on 2 February 2026 and aimed to evaluate the effects of dry needling on structural and mechanical muscle properties as well as neurophysiological measures in individuals with post-stroke spasticity.

### 2.1. Eligibility Criteria for Screening

A structured approach based on predetermined parameters was conducted; see Table 1. Studies were included if they met the following inclusion criteria: (1) Articles written in Dutch, English, French, or Spanish. (2) Both randomized controlled trials and case–control designs were included due to the limited number of available studies investigating the mechanistic outcomes of dry needling in post-stroke spasticity. Given the exploratory nature of the review and the focus on physiological mechanisms rather than solely clinical efficacy, the inclusion of non-randomized designs allowed a broader assessment of structural–mechanical and neurophysiological outcomes. (3) Only studies investigating people with PSS were included. (4) Only studies using dry needling or combining it with other methods were included. (5) Outcomes included measures reflecting SMMPs, defined as parameters representing passive muscle architecture, tissue stiffness, visco-elastic behaviour, or resistance to passive stretch independent of voluntary muscle activation. Eligible measures included ultrasound-derived muscle thickness, fascicle length, and pennation angle; shear wave elastography; myotonometry; and instrumented assessments of passive resistance torque or stiffness. Outcomes included neurophysiological measures reflecting neural reflex excitability or motor unit behaviour. These parameters included the Hoffmann reflex (H-reflex), an electrically evoked response used to assess the excitability of the spinal monosynaptic reflex pathway, and the H/M ratio (H-max/M-max), which represents the ratio between the maximal H-reflex and maximal direct motor response and reflects the relative excitability of the spinal motor neuron pool. Clinical spasticity scales such as the Modified Ashworth Scale (MAS), the Modified Modified Ashworth Scale (MMAS) and the Modified Tardieu Scale (MTS) were seen as spasticity grading outcomes.

### 2.2. Information Sources

The following electronic databases were systematically searched: Web of Science, PubMed, Scopus, and Embase. The final search was conducted on 2 February 2026. The search strategy was adapted for each database to optimize the sensitivity and specificity of retrieval.

### 2.3. Search Strategy

#### 2.3.1. PICOST Framework

The PICOST framework comprising Patient, Intervention, Comparison, Outcome, Study design and Time was utilized as a methodological tool to develop a comprehensive search strategy aimed at effectively addressing the research question [22]. This systematic review focused on identifying eligible studies involving (post-)stroke patients with spasticity (P), dry needling or Dry Needling for Hypertonia and Spasticity (I) and SMMPs together with neurophysiological changes (O). Randomized Clinical Trials and Case–control studies were solely included (S). The search strategy integrated keywords derived from PICOST elements which were linked with Boolean operators. Corresponding Medical Subject Headings (Mesh) in Pubmed and emtree terms in Embase were also used. Additional synonyms and abbreviations were incorporated to enhance coverage. The final search strategies, detailed in Table 2, were refined through an iterative process involving the addition, removal, and adjustment of terms to optimize both the sensitivity and specificity of the search.

#### 2.3.2. Selection Process

All retrieved records were imported into Rayyan (Qatar Computing Research Institute, Doha, Qatar) for duplicate removal and screening [23]. The study selection process was conducted in two stages: (1) title and abstract screening and (2) full-text eligibility assessment. Screening was performed independently by three reviewers (CL, JD, LJ), with each record evaluated by two reviewers. Disagreements were resolved through discussion and, when necessary, consultation with a third reviewer (BE). The reference lists of all the included studies were manually screened to identify additional potentially eligible articles.

#### 2.3.3. Data Collection Process

Prior to data extraction, a standardized extraction form was developed and agreed upon by all reviewers to ensure consistency. Data extraction was performed independently by two reviewers. Any discrepancies were resolved through discussion and, when necessary, consultation with a third reviewer. The extracted data were recorded in a structured evidence table (Table 3) to facilitate comparison and synthesis.

### 2.4. Data Items

The following data items were extracted from each included study: study identification (author, year), study design, participant characteristics, intervention details (including needle size), timing of assessments, outcome measures, and results. The participant characteristics included demographic and clinical variables such as age, sex, and stroke-related features. The intervention characteristics included dry needling parameters and treatment protocols. The primary outcomes were structural–mechanical muscle properties (SMMPs), including measures of passive muscle architecture, tissue stiffness, visco-elastic behaviour, and resistance to passive stretch. The secondary outcomes were neurophysiological measures reflecting neural reflex excitability or motor unit behaviour. Clinical spasticity scales were extracted for descriptive purposes. Neuroinflammatory outcomes were not directly measured but are discussed as a mechanistic framework. The data were extracted at all reported time points (e.g., pre-intervention, post-intervention, and follow-up) when available. When multiple outcome measure time points were reported for the same domain, all relevant data were collected to allow comprehensive comparison across studies. In cases of missing, unclear, or incompletely reported data, attempts were made to extract the most complete information available from the published report. No additional assumptions were made unless explicitly stated.

### 2.5. Risk-of-Bias Assessment

Risk of bias was assessed using the Revised Cochrane Risk-of-Bias tool for randomized trials (RoB2) [24]. Assessments were performed at the outcome level, focusing on the effect of assignment to intervention (intention-to-treat effect). Five domains were evaluated: randomization (D1), deviations from intended interventions (D2), missing outcome data (D3), outcome measurement (D4), and selection of reported results (D5). A synopsis of the risk-of-bias assessment can be found in Table 4.

### 2.6. Effect Measures

To allow comparison across studies reporting outcomes on different measurement scales, treatment effects were expressed as standardized mean differences (SMDs). Effect sizes were first calculated as Cohen’s d and subsequently converted to Hedges’ g to correct for small-sample bias using the correction factor J, where N represents the total sample size. Within-group effects were calculated as standardized mean changes between baseline and post-treatment (T1–T2) and between baseline and follow-up (T1–T3), using the pooled standard deviation of the respective measurements. Between-group effects were estimated using a difference-in-change approach, defined as the difference between the mean change in the intervention group and the mean change in the control group divided by the pooled baseline standard deviation. 

For each effect size, standard errors (SEs) were calculated to estimate the precision of the effect estimates. These g ± SE values were used for all subsequent interpretations. The calculated effect sizes and corresponding standard errors are presented in Table 5. Positive effect sizes were interpreted as favouring the intervention; for outcomes where a decrease reflects improvement (e.g., spasticity grading or ultrasonographic characteristics), the direction of the effect size was interpreted accordingly. The use of standardized effect sizes enabled comparison of treatment effects across heterogeneous outcome measures, including clinical spasticity scales, biomechanical muscle properties, and neurophysiological parameters. Relationships between outcome domains were explored using Pearson correlation coefficients between standardized effect sizes. All statistical analyses were performed using IBM SPSS Statistics (version 31.0.0, IBM Corp., Armonk, NY, USA) [25].

### 2.7. Synthesis Methods

**Figure 1:**
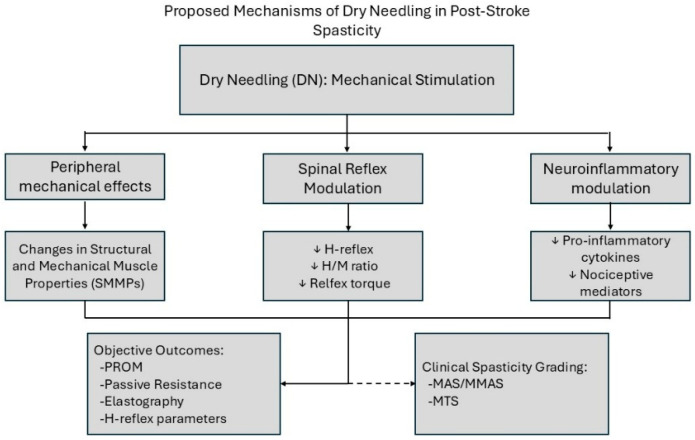
Proposed mechanisms of dry needling (DN) in post-stroke spasticity. DN may induce peripheral mechanical effects, spinal reflex modulation, and neuroinflammatory changes. The symbol (↓) indicates a reduction or decrease in the respective outcome following dry needling. These mechanisms contribute to improvements in objective outcomes such as passive range of motion (PROM), passive resistance, elastography, and H-reflex parameters. The relationship between these objective measures and clinical spasticity grading (MAS/MMAS, MTS) appears weak and inconsistent (dashed arrow).

When studies reported summary statistics in alternative formats (e.g., medians and interquartile ranges), values were converted to approximate means and standard deviations using established statistical conversion methods where appropriate. If the required summary statistics were unavailable, effect sizes were not calculated. A formal meta-analysis was not performed due to (1) substantial heterogeneity in outcome measures, (2) variability in intervention protocols and dosing, (3) differences in study design, and (4) lack of sufficient comparable data across studies. In addition, many studies did not report the necessary variance measures or pre–post correlations required for reliable pooled estimates. Therefore, a structured narrative synthesis combined with standardized effect size estimation was considered more appropriate. To support the interpretation of the findings, a conceptual framework was developed to illustrate the proposed mechanisms underlying the effects of dry needling on post-stroke spasticity. This framework integrates structural–mechanical, neurophysiological, and neuroinflammatory pathways, and their relationship with objective and clinical outcome measures (Figure 1).

### 2.8. Certainty Assessment

The certainty of evidence was assessed using a modified GRADE approach focusing on key domains considered most relevant to the included studies (study limitations, inconsistency, imprecision, overall certainty) [26]. A full GRADE assessment was not performed due to heterogeneity in outcome reporting (Table 6).

**Table 3:** Table of evidence.

Author(s)/Study ID	Patient Characteristics	Intervention	Timetable and Outcome Measures	Results	
Al Amin et al. (2024) [27]	*N* = 90 DN group: *n* = 30 M/F ratio: 19/11 Age (y): 65.30 ± 15.27 Duration of Illness (DOI) (month): 13.62 ± 5.27 ES-group: *n* = 30 M/F ratio: 18/12 Age (y): 62.00 ± 12.56 DOI (month): 12.65 ± 7.70 DN + IMES group: n = 30 M/F ratio: 23/7 Age (y): 60.57 ± 12.97 DOI (month): 13.43 ± 8.94	DN group: ES group: DN + IMES group:	Timetable: Pre- and post-test evaluation Outcomes: -MAS -H-reflex -Maximum latency H-amplitude -M-amplitude -H/M ratio	Immediate post-treatment (single session): H-reflex (neurophysiological outcomes): H/M ratio—gastrocnemius: DN (↓): *p* = 0.024 DN + IMES (↓): *p* = 0.042 ES: no significant change H/M ratio—Soleus: DN (↓): *p* = 0.029 DN + IMES (↓): *p* = 0.001 ES: NS	Maximum wave latency (ms): DN and DN + IMES groups (↑): gastrocnemius and soleus (*p* < 0.01); ES (↑) only for gastrocnemius Spasticity (MAS): MAS score: ES (↓): post-treatment (*p* = 0.002) DN (↓): *p* = 0.0001 DN + IMES (↓): *p* = 0.0001 No between-group differences pre- or post-treatment (*p* > 0.05)
Babazadeh et al. (2024) [28]	First stroke (n = 24) IG: *n* = 12 M/F ratio: 4/8 Mean age: 62.83 (±11.32) DOI (y): 6.33 (±3.44) IG+ exercise therapy (ET): *n* = 12 First-ever unilateral stroke Post-stroke Mean age: 52.50 (±8.60) DOI (y): 9.33 (±6.08)	CG: only DN 4 DN sessions (1 min each muscle) in 4 weeks Deep DN in FCR and FCU IG: DN + ET 4 DN sessions (1 min each muscle) in 4 weeks ET (30 min) each session + repeat ET daily at home	Timetable: -T0 = at baseline -T1 = before intervention -T2 = immediately after intervention -T3 = 3 weeks after last session (follow-up) Outcomes: -PROM and AROM wrist extension -MMAS -H-reflex -ARAT -FMA	Within-group PROM (↑): Time effect: *p* < 0.001 -Post-treatment: *p* = 0.001 -Follow-up: *p* = 0.008 MMAS (↑): DN: *p* = 0.015 DN + ET: *p* = 0.028 -Post-treatment: *p* = 0.025 -Follow-up: *p* < 0.05 AROM (NS): *p* = 0.121 ARAT: DN + ET (↑): *p* = 0.002 DN (NS): *p* = 0.065 FMA (↑): *p* < 0.001 H-reflex latency (↑): *p* = 0.002 Follow-up: *p* = 0.014 Post-treatment: *p* = 0.008 Hmax/Mmax ratio (↑): Time effect: *p* = 0.000033	Between-group PROM (NS): *p* = 0.964 Group-by-time interaction: *p* = 0.39 MMAS (NS): *p* > 0.05 AROM: Group-by-time interaction: DN + ET (↑): *p* = 0.046 DN (NS): *p* > 0.05 ARAT (NS): *p* > 0.05 FMA (NS): *p* = 0.973 H-reflex latency (NS): *p* = 0.51 Hmax/Mmax ratio (NS): *p* = 0.133
Ebrahimi et al. (2024) [29]	*n* = 40 (1:1 group ratio) First stroke ≥6 m duration	CG: resistive and stretching exercises hip ADD, SOL, GC, PNF patterns, gait training, 2×/week, 6 weeks IG: same as CG + DN treatment of hip ADD, TA, SOL, GC, muscle length examination	Timetable: Four sessions: -Before treatment -3 weeks after first session -Immediately after last session -1 week after last session Outcomes: -ROM knee + ankle -MMAS -TUG	Within groups: PROM (knee and ankle ROM) (↑): Time effect: *p* < 0.001 IG: significant improvement from T2 to T4 (*p* < 0.001) CG: = MMAS (↓ spasticity): Time effect: *p* < 0.001 IG: T2-T4 (↓) (*p* < 0.001) CG: slight reduction TUG (↓ time = ↑ function): Time effect: *p* < 0.001 IG: T2-T4 (↓) (*p* < 0.001) CG: T4 (↓)	Between groups: MMAS (↓ spasticity): Group effect significant at: 2nd exam: *p* = 0.023 3rd exam: *p* = 0.002 4th exam: *p* = 0.000 Knee ROM (↑): Significant at 4th exam: *p* = 0.020 Ankle ROM (↑): 3rd exam: *p* = 0.035 4th exam: *p* = 0.004 TUG: No significant between-group differences across examinations
Ghannadi et al. (2020) [30]	*n* = 24 First stroke CG: n = 12 M/F ratio: 7/5 Age (y): 55.9 ± 12.1 Hemiplegic side (left/right): 6/6 DOI (month): 26.4 ± 12.1 IG: *n* = 12 M/F ratio 10/2 Age (y): 58 ± 6.6 Hemiplegic side (left/right): 6/6 DOI (month): 23.9 ± 13.2	IG: 3 DN sessions in 1 week. At least 48 h between treatment sessions CG: sham DN	Timetable: -T0 = at baseline -T1 = immediately after 3rd session of DN (1 week) -T2 = after 1 month Outcomes: Primary: -MMAS -TUG -10 m walk test) Secondary: -Ankle AROM extension -Ankle PROM extension -Single-leg stance test -Barthel index -Pennation angle -GC muscle thickness	MMAS Group-by-time interaction: *p* < 0.001 -IG: ↑ -CG: no change Time effect: *p* < 0.001 TUG Group-by-time interaction: *p* < 0.001 -IG: ↑ -CG: no change 10-MWT Group-by-time interaction: *p* = 0.02 -IG: ↑ -CG: no change SLS test Group-by-time interaction: *p* < 0.001 -IG: ↑ (*p* < 0.001) -CG: no change AROM Group-by-time interaction: *p* = 0.658 -IG: no change	-CG: no change PROM Group-by-time interaction: *p* < 0.001 -IG: --T0–T1 (↑): *p* < 0.001 --T1–T2 (NS): *p* = 0.723 -CG: no change BI index Group-by-time interaction: *p* < 0.001 -IG: ↑ -CG: no change Pennation angle Group-by-time interaction: *p* < 0.001 -IG: ↑ -CG: no change Muscle thickness Group-by-time interaction: *p* < 0.001 IG: ↑ CG: no change
Kamble et al. (2024) [31]	*N* = 81 IG: *n* = 41 CG: *n* = 40	IG: 6× DN sessions in 2 weeks with conventional therapy CG: only conventional therapy (2 w)	Timetable: -Pre-test -Post-test -Follow-up (after 4 weeks) Outcomes: -H-reflex MTS	Modified Tardieu Scale (MTS): Within DN group: *p* < 0.001 Between groups (Δ pre–post): *p* = 0.003 Between groups (Δ pre–follow-up): *p* < 0.001	H-reflex: Within DN group: *p* < 0.001 Between groups (Δ pre–post): *p* = 0.004 Between groups (Δ pre–follow-up): *p* = 0.001 Follow-up (4 weeks): Between-group differences for MTS and H-reflex remained significant (*p* < 0.05)
Kösem et al. (2022) [32]	*N* = 30 BTX-A group = 15 M/F: 11/4 Age (med and min/max): 59 y (46/79) Time since onset (month): 37 Hemiplegic side (left/right): 5/10 BTX-A + DN group = 15 M/F 9/6 Age (med and min/max): 64 y (28/78) Time since onset (month): 23 Hemiplegic side (left/right): 8/7	BTX: 1×500U (200U BB) + 45 min Exercise programme BTX+ DN: 1×500U (200U BB) + 4× DN session in 2 w +45 min Exercise program	Timetable: -Before treatment (BT) -Immediately after treatment (IAT) -Third day after treatment (AT3) -Second week after treatment (AT2W) -Third month after treatment (AT3M) Outcomes: -MAS -MTS -FMA	Post-treatment (BTX + DN vs. BTX): MAS (↓ spasticity): Median 1 (1–2) vs. 2 (2–3), *p* < 0.05 Modified Tardieu Scale (MTS): V1X: 1 (0–2) vs. 2 (1–2), *p* < 0.05 V3X: 2 (1–3) vs. 3 (2–4), *p* < 0.05 V3Y (°): 100 (60–110) vs. 70 (32–87), *p* < 0.05 Dynamic contracture angle (°): 40 (30–80) vs. 70 (53–108), *p* < 0.05	Upper-extremity motor function (FMMFS): 37 (20–48) vs. 24 (10–47), *p* < 0.05 Follow-up (3 months): Between-group differences in MAS, MTS parameters, dynamic contracture, and FMMFS remained significant (*p* < 0.05)
Kucuktepe et al. (2023) [33]	*n* = 42 First-ever stroke ≥6 m duration CG: *n* = 21 M/F ratio: 13/8 Age (y): 62.86 ± 8.97 Hemiplegic side (left/right): 3/18 DOI (month): 2.83 ± 3.46 IG: *n* = 21 M/F ratio: 10/11 Age (y): 63.57 ± 7.76 Hemiplegic side (left/right): 9/12 DOI (month): 2.38 ± 1.49	CG: NDT: 4 weeks, 3×/week, 45 min/day (12 sessions) IG: NDT + DN: DN (~60 s/muscle) + NDT (12 sessions)	Timetable: -T1 = at baseline -T2 = after 1st session -T3 = post-intervention Outcomes: Primary: -ROM -MAS Secondary: -NHPT -10-MWT -FAB	Within groups IG MAS: -GC (↑): *p* = 0.025 -QF (NS): *p* = 0.174 -FCR (NS): *p* = 0.405 -BB (NS): *p* = 0.075 AROM (↑) -Knee flex: *p* < 0.001 -Ankle dfl: *p* < 0.001 -Elbow ext: *p* < 0.001 -Wrist ext: *p* < 0.001 PROM -Knee flex (↑): *p* < 0.001 -Ankle dfl (↑): *p* < 0.001 -Elbow ext (↑): *p* < 0.001 -Wrist ext (↑): *p* < 0.001	Between groups AROM (↑): -Knee flex: *p* = 0.001 -Ankle dfl: *p* < 0.001 -Elbow ext: *p* < 0.001 -Wrist ext: *p* < 0.001 PROM -Knee flex (↑): *p* < 0.001 -Ankle dfl (↑): *p* = 0.001 -Elbow ext (↑): *p* = 0.002 -Wrist ext (NS): *p* = 0.268 NHPT (↑) Paretic side: *p* < 0.001 Non-paretic side: *p* = 0.011 10-MWT (↑): *p* < 0.001 FAB (↑): *p* < 0.001
Nakhostin Ansari et al. (2023) [34]	*N* = 24 (ratio 1:1) First-ever stroke ≥6 m duration M/F ratio: 13/11 Age (y): 59 ± 12.0 Hemiplegic side (left/right): 16/8 DOI (years): Group 1: 28.92 ± 28.72 Group 2: 24.58 ± 12.22	Group 1: one session DN of FCR and FCU Group 2: three sessions DN of FCR and FCU	Timetable -T0 = before DN -T1 = immediately after DN -T2 = one week after DN Outcomes: -MMAS -PRT -PROM wrist -AROM wrist -Motor recovery (BSSR)	Within-group results: MMAS: -Group 1 (↑): *p* < 0.001 -Group 2 (↑): *p* < 0.001 --T1–T2 (↓): *p* = 0.41 PRT (↑): -Group 1: no change -Group 2 (↑): *p* < 0.001 Time-by-group interaction (↑): *p* = 0.02 Time effect (↑): *p* < 0.001 --T1–T2 (NS): *p* = 1.0 PROM wrist (↑): -Group 1: *p* < 0.001 -Group 2: *p* < 0.001 Time-by-group interaction (↑): *p* = 0.02 Time effect (↑): *p* < 0.001 --T1–T2 (NS): *p* = 0.82	AROM wrist (↑): -Group 1: *p* < 0.001 -Group 2: *p* < 0.001 Time-by-group interaction (↑): *p* = 0.001 Time effect (↑): *p* < 0.001 --T1–T2 (NS): *p* = 1.0 BSSR (↑): -Group 1: *p* < 0.001 -Group 2: *p* < 0.001 --T1–T2 (NS): *p* = 0.31 Results between groups (↓): MMAS: *p* > 0.05 PRT: *p* = 0.28 PROM wrist: *p* = 0.06 AROM wrist: *p* = 0.2 BSSR: *p* > 0.05
Panahi et al. (2024) [35]	*n* = 24 IG: n = 12 Age: 55.08 (9.56) M/F ratio: 4/8 Affected limb L/R: Disease duration: 15.42 (4.83) SCG: *n* = 12 Age: 53.92 (9.71) M/F ratio: 6/6 Affected limb L/R: Disease duration: 14.08 (5.57)	IG: 12 weeks of neurorehabilitation + 4× DN SCG: 12 weeks of neurorehabilitation + 4× sham DN	Timetable: -T0 = pre-test -T1 = post-test -T2 = follow-up (one month later) Outcomes: -DUS -MAS -FMA -B and B -Reflex torque (isokinetic)	Within-group results: IG Muscle thickness ↓ significant (*p* < 0.01) Reflex torque ↓ significant (*p* < 0.01) MMAS ↓ significant (*p* < 0.01) FMA-UE ↑ significant (*p* < 0.01) BBT ↑ significant (*p* < 0.01)	Between groups: IG Muscle thickness (*p* < 0.01) Reflex torque (*p* < 0.01) MMAS No significant difference FMA-UE (*p* < 0.01) BBT (*p* < 0.01)
Parsaei et al. (2025) [36] Case–control	*N* = 20 M/F: 14/6 Age: 55.3 ± 9.50 Time since onset: 15.5 ± 11.4 (months)	Single-group sham-controlled trial First: 3× 1 w sham needling + Second: wash-out week Third: 3× 1 w dry needling SN and DN: Pronator muscles arm	Timetable: -T0 = pre-test sham -T1 = post-test sham -T2 = pre-test DN -T3 = post-test DN -T4 = follow-up (one week later) Outcomes: -AROM (elbow extension/supination, wrist extension) -PROM -MMAS -BSSR -CAHAI-13	Within-group results: MMAS (↓): Significant reduction in forearm pronators and wrist flexors (*p* < 0.001); no change in elbow flexors AROM and PROM (↑): Elbow extension, forearm supination, and wrist extension improved (*p* < 0.01) Motor recovery (BSSR) (↑): Improved (*p* < 0.001) Upper-limb function (CAHAI-13) (↑): Improved (*p* < 0.01; <MCID) Sham period: No significant changes (*p* > 0.05)	Results between conditions: Greater improvements in MMAS, AROM, PROM, and BSSR (*p* < 0.01–0.001) Follow-up: Improvements in MMAS, AROM, PROM, and BSSR maintained; CAHAI-13 remained statistically but not clinically improved
Tavakol et al. (2021) [37]	*n* = 24 (1:1 group ratio) M/F ratio: 17/7 Mean age (y): 57.0 ± 9.6 IG: DN (n = 12) CG: sham DN (n = 12) Hemiplegia: first-ever stroke; ≥6 m duration	3 sessions, separated by a 48 h interval between sessions Targeted muscles: -FCR -FCU	Timetable: -T0 = pre-test -T1 = post-test -T2 = follow-up (4 weeks later) Outcomes: Primary: -MMAS -BBT Secondary: -AROM -PROM wrist	Within-group results: IG MMAS (↑): *p* < 0.001 BBT (NS): -Group-by-time interaction: *p* = 0.187 -Time effect: *p* = 0.421 AROM wrist (NS) -Group-by-time interaction: *p* = 0.145 -Time effect: *p* = 0.311 PROM wrist (↑) -Group-by-time interaction: *p* < 0.001	-Time effect: *p* < 0.001 CG MMAS (NS): *p* = 0.37 Between-group results: MMAS (↑): *p* = 0.012 BBT (NS): *p* = 0.244 AROM wrist (NS): *p* = 0.249 PROM wrist (↑): *p* = 0.040
Zhang et al. (2021) [38]	*n* = 210 DN: n = 70 M/F ratio: 47/23 Age (y): 66.17 ± 9.84 Hemiplegic side (left/right): 34/36 DOI (month): 12.67 ± 3.09 SDN: *n* = 70 M/F ratio: 44/26 Age (y): 62.97 ± 11.53 Hemiplegic side (left/right): 33/37 DOI (month): 13.41 ± 2.98 CG: *n* = 70 M/F ratio: 48/22 Age (y): 65.07 ± 8.5 Hemiplegic side (left/right): 37/33 DOI (month): 12.54 ± 3.04	DN: DN at MTP treatment 5 times, 30 min each time, every week for 4 weeks + routine rehab treatment SDN: DN + adjacent area MTP treatment 5 times, 30 min each time, ever week for 4 weeks + routine rehab treatment CG: routine rehab treatment 5 times every week, 4 weeks + routine anti-stroke treatment	Timetable: -At baseline -After each treatment -After 4 weeks Outcomes: Primary: -MAS -Spasticity relief thumb Secondary: -Spasticity relief thumb and fingers 2-5 -PROM thumb, wrist and fingers 2-5	Primary: MAS: -DN (↑): *p* < 0.001 -DN > SDN/control (↑): *p* < 0.05 -SDN–control: *p* > 0.05 Spasticity relief thumb -DN: *p* < 0.05 Secondary: Spasticity relief thumb -DN: *p* < 0.05 -DN> SDN/control: *p* < 0.05 -SDN–control: *p* > 0.05 Spasticity relief fingers 2-5 -DN: *p* < 0.05 -DN> SDN/control: *p* < 0.05 -SDN–control: *p* > 0.05 MAS: baseline—4 weeks Fingers: -DN: *p* = 0.018 -DN > SDN/control: *p* < 0.05 -SDN–control: *p* > 0.05 Wrist: -DN: *p* = 0.013 -DN > SDN/control: *p* < 0.05 -SDN–control: *p* > 0.05	Thumb: -DN: *p* = 0.012 -DN > SDN/control: *p* < 0.05 -SDN–control: *p* > 0.05 Changes joint angles of hand in rest position Wrist: -DN: *p* = 0.047 -DN–SDN/control *p* > 0.05 -SDN–control: *p* > 0.05 MCP thumb: -DN: *p* = 0.122 -DN–SDN/control: *p* > 0.05 -SDN–control: *p* > 0.05 IP thumb: -DN: *p* = 0.031 -DN–SDN/control: *p* > 0.05 -SDN–control: *p* > 0.05 MCP fingers 2-5: -DN: *p* = 0.173 -DN–SDN/control: *p* > 0.05 -SDN–control: *p* > 0.05 PIP fingers 2-5: -DN: *p* = 0.018 -DN > SDN: *p* < 0.05 -DN-control: *p* > 0.05 -SDN–control: *p* > 0.05 DIP fingers 2-5: -DN: *p* = 0.081 -DN–SDN/control: *p* > 0.05 -SDN–control: *p* > 0.05

ADDs: adductors; ARAT: action research arm test; AROM: active range of motion; BB: biceps brachii; BBT: box and block test; BI index: Barthel index; BSSR: Brunnstrom stages of stroke; CAHAI-13: Chedoke arm and hand activity inventory; CG: control group; CHS: cerebral haemorrhagic stroke; CIS: cerebral ischemic stroke; dfl: dorsiflexion; DIP: distal interphalangeal joint; DN: dry needling; DOI: duration of illness; ES: electric stimulation; ET: exercise therapy; FAB: Fullerton advanced balance scale; FCR: flexor carpi radialis muscle; FCU: flexor carpi ulnaris muscle; FMA: Fugl–Meyer assessment; GC: gastrocnemius; GF: quadriceps (vastus medialis); GL: gastrocnemius lateralis; GM: gastrocnemius medialis; IG: intervention group; IMES: intramuscular electrical stimulation; IP: interphalangeal joint; MAS: modified Ashworth scale; MCP: metacarpophalangeal joint; MMAS: modified modified Ashworth scale; MTP: myofascial trigger point; NDT: neurodevelopment therapy; NHPT: hole peg test; PIP: proximal interphalangeal joint; PRF: passive resistance force; PROM: passive range of motion; PRT: passive resistance torque; ROM: range of motion; SDN: sham dry needling; SLS test: single-leg stance test; SOL: soleus; SS: static stretching; TA: tibialis anterior; TUG: timed up-and-go test; US: ultrasonographic; 10-MWT: 10-metre walk test; ↑: significance (*p* < 0.05)/improvement; ↓:reduction in grading or score; NS: no significance (*p* > 0.05)/no improvement; >: effective rate was higher.

**Table 4:** Risk-of-bias assessment (RoB 2).

Study	D1	D2	D3	D4	D5	Overall
Al Amin et al. (2023) [27]	Low	SC	Low	SC	Low	Some concerns
Babazadeh et al. (2024) [28]	Low	SC	Low	Low	Low	Some concerns
Ebrahimi et al. (2024) [29]	Low	SC	Low	SC	Low	Some concerns
Ghannadi et al. (2020) [30]	SC	Low	Low	Low	Low	Some concerns
Kamble et al. (2024) [31]	High	High	Low	High	Low	High
Kosem et al. (2022) [32]	Low	Low	Low	Low	Low	Low
Kucuktepe et al. (2023) [33]	Low	Low	Low	Low	Low	Low
Nakhostin Ansari et al. (2023) [34]	Low	Low	Low	Low	Low	Low
Panahi et al. (2024) [35]	Low	Low	Low	Low	Low	Low
Parsaei et al. (2025) [36]	High	High	Low	SC	Low	High
Tavakol et al. (2021) [37]	Low	Low	Low	Low	Low	Low
Zhang et al. (2021) [38]	Low	Low	Low	Low	Low	Low

Domains: D1: Bias arising from the randomization process. D2: Bias due to deviations from intended intervention. D3: Bias due to missing outcome data. D4: Bias in measurement of the outcome. D5: Bias in selection of the reported result.

**Table 5:** Effect size calculation. (**A**) Structural and mechanical muscle property outcomes. (**B**) Neurophysiological outcomes.

(A)				
Study	Outcome	Comparison	Post-Treatment (g)	Follow-Up (g)
Babazadeh-Zavieh et al. (2024) [28]	PROM Wrist Extension (°)	DN	0.23	0.21
	PROM Wrist Extension (°)	DN + ET	0.65	0.42
	PROM Wrist Extension (°)	Between-group	0.20	0.05
Ebrahimi A. et al. (2024) [29]	Knee PROM	DN + ET	1.11	1.21
	Knee PROM	ET	0.01	0.03
	Knee PROM	Between-group	1.12	1.16
Ebrahimi A. et al. (2024) [29]	Ankle PROM	DN + ET	1.79	1.68
	Ankle PROM	ET	0.54	0.10
	Ankle PROM	Between-group	1.01	1.37
Ghannadi et al. (2020) [30]	Ankle PROM	DN	0.67	0.77
	Ankle PROM	Sham DN	−0.04	0.06
	Ankle PROM	Between-group	0.87	0.86
Ghannadi et al. (2020) [30]	Pennation Angle	DN	−1.29	−1.26
	Pennation Angle	Sham DN	0.83	0.01
	Pennation Angle	Between-group	2.37	1.57
Ghannadi et al. (2020) [30]	Muscle Thickness	DN	−1.88	−1.82
	Muscle Thickness	Sham DN	−0.06	−0.04
	Muscle Thickness	Between-group	2.08	2.08
Kucuktepe et al. (2023) [33]	Knee Flexion PROM	DN + NDT	NA	0.44
	Knee Flexion PROM	NDT	NA	0.40
	Knee Flexion PROM	Between-group	NA	0.03
Kucuktepe et al. (2023) [33]	Ankle Dorsiflexion PROM	DN + NDT	NA	0.92
	Ankle Dorsiflexion PROM	NDT	NA	0.35
	Ankle Dorsiflexion PROM	Between-group	NA	0.35
Kucuktepe et al. (2023) [33]	Elbow Extension PROM	DN + NDT	NA	−0.49
	Elbow Extension PROM	NDT	NA	−0.73
	Elbow Extension PROM	Between-group	NA	−0.33
Kucuktepe et al. (2023) [33]	Wrist Extension PROM	DN + NDT	NA	0.69
	Wrist Extension PROM	NDT	NA	0.81
	Wrist Extension PROM	Between-group	NA	0.03
Nakhostin Ansari et al. (2023) [34]	Wrist PROM	Intervention 1x	0.28	0.27
	Wrist PROM	Intervention 3x	1.09	0.69
	Wrist PROM	Between-group	0.30	0.27
Nakhostin Ansari et al. (2023) [34]	PRT	Intervention 1x	−0.17	−0.17
	PRT	Intervention 3x	−0.66	−0.69
	PRT	Between-group	0.29	0.37
Tavakol et al. (2020) [37]	Wrist PROM	DN	3.05	3.10
	Wrist PROM	Control sham DN	0.02	0.03
	Wrist PROM	Between-group	2.11	2.14
(**B**)				
Al Amin et al. (2023) [27]	H/M Ratio (Soleus + GM)	DN	0.58	0.58
	H/M Ratio (Soleus + GM)	DN + IMES	0.67	0.48
	H/M Ratio (Soleus + GM)	ES	0.42	0.40
Kamble et al. (2024) [31]	H-reflex	Intervention	0.94	1.01
	H-reflex	Control	0.84	0.60
	H-reflex	Between-group	0.73	NA
	MTS	Intervention	0.46	1.03
	MTS	Control	0.12	0.36
	MTS	Between-group	0.63	NA

Notes. All standardized effects in this table are reported as Hedges’ g (converted from Cohen’s d using the small-sample correction factor J). Positive values indicate improvement in favour of the intervention. Very large effect sizes (g > 2.0) should be interpreted with caution, as they may reflect small sample sizes or variability rather than true clinical magnitude. for outcomes where a decrease reflects improvement, effect sizes were directionally interpreted accordingly. Negative values therefore indicate numerical change direction, not necessarily clinical worsening. For Panahi et al. (2024) [35], Parsaei et al. (2025) [36] and Zhang et al. (2021) [38], effect size calculations were not possible due to insufficient data. All post-treatment comparisons refer to baseline versus the assessment immediately after the final intervention.

**Table 6:** Certainty of evidence (GRADE-scoring) table.

Outcome Domain	Risk of Bias	Inconsistency	Indirectness	Imprecision	Publication Bias	Overall Certainty
Spasticity grading (MAS/MMAS)	Serious	Serious	Not serious	Serious	Undetected	Low
Structural and mechanical muscle properties (PROM, passive resistance, muscle thickness)	Not serious	Serious	Not serious	Serious	Undetected	Moderate
Modified Tardieu Scale (MTS)	Not serious	Not serious	Not serious	Serious	Undetected	Moderate
Neurophysiological outcomes (H-reflex, H/M ratio, reflex torque)	Serious	Serious	Not serious	Serious	Undetected	Low

## 3. Results

### 3.1. Search Strategy

**Figure 2:**
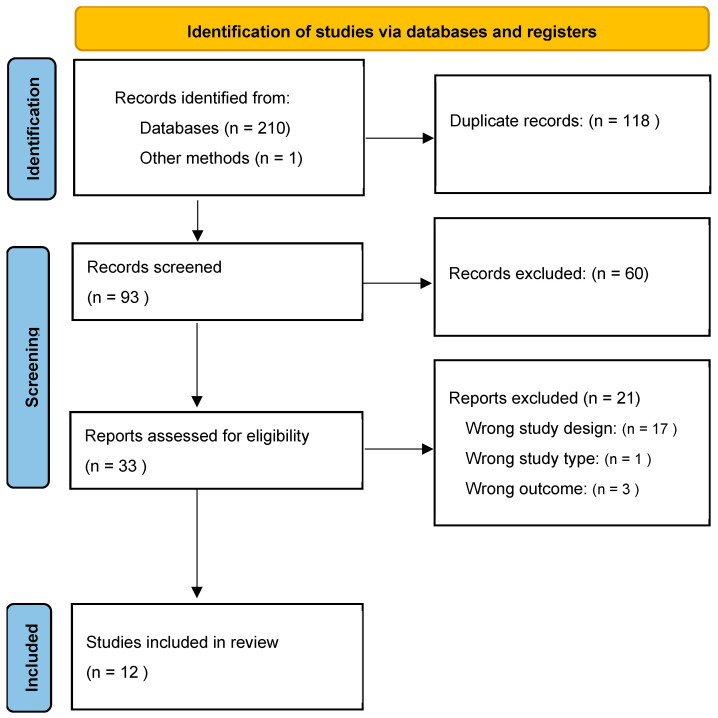
PRISMA flow diagram illustrating the study selection process for the systematic review.

The final search strategy yielded 226 records. After the removal of duplicates and screening of titles and abstracts, 12 studies met the eligibility criteria and were included in this review. The study selection process is illustrated in Figure 2, and the study characteristics are summarized in Table 3.

### 3.2. Demographic Characteristics

Sample sizes ranged from 20 to 90 participants, with one study including 210 participants across three groups. The pooled mean age was 59.2 years, with a reported sex distribution of 324 males and 188 females. The mean time since stroke was 5.16 years. In eight of the twelve included studies, participants were more than six months post-stroke, indicating that most samples represented chronic stroke populations.

### 3.3. Study Characteristics and Interventions

Seven of the twelve studies included both an intervention and a control group, of which four used sham DN as the comparator [29,31,33,35,36,37,38]. Five studies did not include a traditional control group; one compared two DN dosages, one added BTX treatment as an adjacent intervention, and another used a within-subject sham-controlled design [27,28,30,32,34]. The number of DN sessions varied considerably, ranging from a single session to 20 sessions. Intervention duration ranged from one day to five weeks. In most studies, DN was administered two to three times per week, with an approximate treatment time of one minute per muscle. One study did not report the exact application time. Nine studies applied a fast-in fast-out technique, whereas one study used an adjacent needling approach with needle retention for 30 min.

### 3.4. Targeted Muscles

DN was most frequently applied to the wrist and finger flexor muscles, particularly the flexor carpi radialis and flexor carpi ulnaris [28,34,35,36,37]. The gastrocnemius was the most targeted lower-limb muscle, with two studies targeting both the medial and lateral heads [27,30]. The other muscles targeted included the soleus, tibialis anterior, hip adductors, and forearm pronators.

### 3.5. Outcomes

The results are reported separately for the within-group changes and between-group comparisons. Within-group improvements reflect changes over time within a single group, whereas between-group differences provide a more robust estimate of treatment efficacy.

#### 3.5.1. Structural–Mechanical Muscle Properties (SMMPs)

Eight studies evaluated structural–mechanical outcomes, including passive range of motion (PROM), passive resistance torque, and ultrasound-derived muscle architecture [28,29,30,33,34,36,37,38]. Overall, DN produced consistent improvements in mechanical muscle properties, with more robust findings than those observed for neurophysiological outcomes.

Within-group changes

All eight studies assessing PROM reported significant increases following DN, indicating improved joint mobility and reduced passive stiffness. Two studies using ultrasonography observed increases in muscle thickness and pennation angle, suggesting favourable adaptations in muscle architecture [30,35]. One study measuring passive resistance torque demonstrated a reduction in torque during slow passive stretch, with larger improvements following multi-session DN protocols compared with single-session interventions [34].

Between-group differences

Between-group effects were more variable. Several trials reported greater PROM improvements in DN groups compared with sham or conventional therapy, although these differences were not universal across joints or studies. Ultrasound-based measures showed clearer between-group differences favouring DN, whereas passive torque findings were limited by the small number of controlled studies. In some trials, within-group improvements did not translate into statistically significant between-group differences, reflecting heterogeneity in protocols and outcome sensitivity.

Magnitude and interpretation of effects

Structural–mechanical outcomes generally demonstrated larger standardized effect sizes than neurophysiological or clinical spasticity measures. However, interpretation of these values requires caution. Many studies lacked complete variance data or pre–post correlations, which can inflate standardized effect estimates, particularly in small samples. Despite these limitations, the overall pattern across studies indicates that DN reliably improves mechanical muscle properties, with multi-session protocols producing the most consistent effects.

Relationship with clinical spasticity

Improvements in SMMPs did not consistently correlate with reductions in clinical spasticity grading (MAS/MMAS). PROM gains were often substantial even when changes in spasticity scores were modest or absent. This suggests that mechanical adaptations—such as reduced stiffness or improved muscle extensibility—may occur independently of neural changes captured by clinical scales, highlighting the multidimensional nature of post-stroke spasticity.

#### 3.5.2. Neurophysiological Effects

Five studies evaluated neurophysiological outcomes, primarily using H-reflex parameters, H/M ratio, reflex torque, and Modified Tardieu Scale (MTS) measures [27,28,31,32,35]. Overall, DN produced moderate reductions in spinal reflex excitability, although findings were less consistent than those observed for structural–mechanical outcomes.

Within-group changes

Most studies reported decreases in H-reflex amplitude or H/M ratio following DN, indicating reduced excitability of the monosynaptic reflex pathway (d ≈ 0.6–1.0). These reductions were generally moderate in magnitude and most evident in protocols involving multiple DN sessions. Reflex torque, assessed in one study, also decreased following DN (d ≈ 3.0), suggesting reduced velocity-dependent resistance during passive stretch [35]. Improvements in dynamic spasticity (MTS R1 angle and dynamic contracture d ≈ 0.7–1.2) were observed in several studies [31,32].

Between-group differences

Between-group effects were less consistent. Trials comparing DN with sham or conventional therapy reported significant reductions in H-reflex parameters in some cases, but not uniformly across studies. When present, between-group differences tended to be smaller than within-group changes, reflecting the limited number of studies with adequate control conditions and the variability in neurophysiological measurement protocols.

Magnitude and interpretation of effects

Although some studies reported large standardized effect sizes, these values should be interpreted cautiously. Many trials lacked pre–post correlation coefficients or complete variance data, which can inflate standardized effect estimates, particularly in small samples. Despite this limitation, the overall pattern across studies suggests that DN can modulate spinal reflex excitability, but the magnitude and durability of these effects remain uncertain.

Relationship with clinical spasticity

Across studies reporting both neurophysiological and clinical outcomes, reductions in H-reflex measures showed a modest association with improvements in spasticity grading, though this relationship did not reach statistical significance. This suggests that neurophysiological adaptations may contribute to—but do not fully explain—clinical changes in spasticity.

### 3.6. Certainty Assessment (Table 6)

Evidence certainty varied across outcome domains. For spasticity grading (MAS/MMAS) several studies presented some concerns or high risk of bias, particularly in deviations from intended interventions and outcome measurement. Combined with heterogeneity in effect sizes and attenuation at follow-up, the certainty of evidence was rated as low. For structural and mechanical muscle properties (SMMPs), including PROM, passive resistance, and muscle thickness, effects were more consistent and generally larger. Most trials were judged at low risk of bias, although clinical heterogeneity and modest sample sizes introduced imprecision, resulting in moderate certainty. Evidence for the Modified Tardieu Scale was also judged as moderate certainty, while neurophysiological outcomes were limited by small samples and methodological variability, leading to low certainty. Overall, mechanical adaptations appear more consistently supported than neural modulation.

### 3.7. Risk-of-Bias Assessment (Table 4)

Most parallel-group randomized trials demonstrated adequate sequence generation and balanced baseline characteristics and were therefore judged as low-risk in D1. However, incomplete reporting of allocation concealment in some studies led to ratings of some concerns, and crossover studies Parsaei et al. (2025) [36] or less rigorously described designs were judged as high-risk. Performance bias was a recurring concern. Given the invasive nature of dry needling, participant and provider blinding was often not feasible. Several studies were therefore rated as having some concerns in D2, and high risk was assigned where co-interventions or adherence were insufficiently controlled. This domain was particularly relevant for clinically assessed outcomes. The missing outcome data were minimal and generally balanced between groups, resulting in predominantly low risk ratings for D3. In contrast, measurement bias (D4) varied by outcome type. Assessor-dependent measures such as MAS/MMAS and manual goniometry were more vulnerable to detection bias in the absence of blinded assessment. Instrument-based measures (Tardieu parameters, H-reflex, ultrasonography) were less susceptible but remained dependent on standardized procedures. The selective reporting bias (D5) was judged as low in most trials, with no clear evidence of outcome switching. Overall, most conventional RCTs were judged at low risk of bias, whereas higher risk ratings were concentrated in open-label or crossover designs. Notably, studies with higher risk-of-bias ratings tended to report larger and less stable effect sizes, suggesting potential inflation of treatment effects in methodologically weaker trials. Across studies, the most frequent sources of bias were (1) lack of participant/provider blinding (D2), (2) assessor-dependent outcome measurement (D4), and (3) small sample sizes. These factors are known to inflate standardized effect sizes, particularly in subjective scales such as MAS/MMAS. In contrast, instrument-based outcomes (H-reflex, reflex torque, ultrasound-derived measures) were less vulnerable to detection bias and are therefore considered methodologically more robust. Notably, trials rated as having higher risk of bias tended to report larger and less stable effect sizes, suggesting potential small-sample and performance-related inflation.

## 4. Discussion

### 4.1. Interpretation of Effects

This review aimed to determine whether DN produces measurable changes in SMMPs in PSS and whether these changes are proportionally reflected in clinical spasticity grading. The aggregated evidence suggests that DN appears to primarily exert peripheral mechanical effects, demonstrated by consistent and comparatively larger improvements in SMMPs, particularly PROM and passive mechanical parameters. These findings support reduced muscle stiffness and increased visco-elastic compliance as the dominant mechanism [18]. Across studies, DN was associated with moderate-to-very-large standardized effects on SMMPs, particularly PROM (g 0.2 to >2.0; although larger values should be interpreted cautiously due to potential methodological inflation). The absence of a significant correlation between mechanical improvements and MAS/MMAS reductions (r = 0.16) suggests that increased joint mobility is not linearly explained by changes in clinical spasticity scores. One possible explanation is that DN induces short-term alterations in local muscle stiffness and visco-elastic behaviour, which may directly influence the mechanical outcomes assessed [39].

### 4.2. Neural Findings

Moderate reductions in H-reflex amplitude, H/M ratio, and reflex torque suggest partial modulation of spinal reflex excitability, suggesting that DN may exert limited central neuromodulatory effects. In addition to these neural mechanisms, emerging evidence suggests that dry needling may also influence neuroinflammatory processes. Several studies suggest that DN may modulate local inflammatory responses, including reductions in pro-inflammatory cytokines and nociceptive mediators [16,40]. These changes may contribute to alterations in muscle stiffness, pain sensitivity, and neural excitability, thereby providing an additional pathway through which DN may affect spasticity. This supports the concept that DN exerts multifactorial effects, involving peripheral mechanical, neural, and inflammatory mechanisms. The association between neurophysiological measures and spasticity grading (r = 0.45) was stronger than that observed between SMMPs and spasticity grading, although it did not reach statistical significance and should therefore be interpreted cautiously given the limited number of studies. Importantly, reductions in MAS/MMAS scores were only weakly aligned with neurophysiological changes, indicating that clinical tone grading may insufficiently capture underlying neural adaptations.

### 4.3. Methodological Limitations

Substantial heterogeneity was present across studies concerning intervention duration, treatment frequency, adjunct therapies, stroke chronicity, and baseline spasticity severity. Baseline MAS/MMAS scores were frequently low (e.g., median 1 in some studies), introducing potential floor effects that may limit observable reductions in clinical spasticity. Additionally, some studies reported exceptionally large PROM effects (g > 2.0), which may partly reflect statistical inflation related to small standard deviations or baseline variability rather than purely clinical magnitude. Most importantly, many included trials had relatively small sample sizes (approximately 12–21 participants per arm), increasing variance and the risk of effect size overestimation despite the use of Hedges’ correction. An important limitation relates to the absence of reported pre–post correlations in most included studies. As a result, standardized mean changes were calculated using pooled standard deviations rather than paired estimates, which may lead to overestimation of effect sizes, particularly in studies reporting very large effects.

### 4.4. Clinical Implications

The observed mechanistic dissociation between mechanical and neural outcomes has important clinical implications. Improvements in joint mobility following DN appear to primarily reflect peripheral mechanical changes, such as reduced passive resistance and altered visco-elastic muscle behaviour. These changes may be clinically meaningful for improving mobility and facilitating movement, even when reductions in reflex hyperexcitability are limited. Conversely, the modest neurophysiological effects observed suggest that DN may contribute to partial modulation of spinal excitability, but these adaptations are not consistently captured by commonly used clinical grading scales such as MAS/MMAS. This reinforces longstanding concerns regarding the construct validity of these scales, particularly their limited ability to distinguish neural from non-neural contributors to resistance during passive movement. Overall, the findings suggest that DN may be most effective as an intervention targeting peripheral mechanical contributors to post-stroke spasticity, while clinical assessment strategies should incorporate outcome measures capable of distinguishing mechanical from neural components.

### 4.5. Future Research

Future research should prioritize several methodological improvements. First, studies should implement standardized DN protocols with clearly defined dosing parameters, including treatment frequency, number of sessions, and targeted muscles. Furthermore, adequately powered randomized controlled trials with blinded assessors are needed to reduce bias and improve the precision of effect estimates. In addition, future studies should incorporate multimodal assessment frameworks, simultaneously evaluating SMMPs (PROM, visco-elastic properties such as stiffness and torque, and imaging-based muscle morphology), neurophysiological measures (H-reflex parameters, reflex torque), and clinical grading (MTS) within the same cohort. Such designs would help clarify the relative contributions of peripheral mechanical adaptations and central neuromodulation. Finally, research should incorporate functional outcomes related to activity and participation to determine whether observed mechanical or neurophysiological changes translate into clinically meaningful improvements. Stratification by stroke chronicity and baseline spasticity severity may further help identify the patient subgroups most likely to benefit from DN. Establishing a clearer mechanistic framework distinguishing peripheral from central effects will be essential for optimizing DN application and guiding evidence-based clinical decision-making in post-stroke spasticity management.

## 5. Conclusions

Dry needling appears to produce measurable changes in structural and mechanical muscle properties in individuals with PSS, with consistent improvements in passive range of motion, muscle stiffness, and ultrasound-derived morphology. Neurophysiological outcomes suggest moderate reductions in spinal reflex excitability, although the certainty of evidence is lower due to methodological variability and small sample sizes. Overall, the findings indicate that DN appears to predominantly influence peripheral mechanical mechanisms, with secondary and less consistent effects on neural pathways. Clarifying the relative contribution of these mechanisms is essential for mechanism-informed clinical applications. Further high-quality studies integrating structural–mechanical, neurophysiological, and functional outcomes are required to clarify optimal clinical application within post-stroke spasticity management.

## Figures and Tables

**Table 1:** Inclusion and exclusion criteria.

	Inclusion	Exclusion
Language	- Dutch - English - French - Spanish	- Other languages
Population, patient, characteristics	- Stroke - Spasticity due to stroke - Post-stroke spasticity (PSS) - Stroke patients - Post-stroke patients - CVA patients - Muscle spasticity - Stroke rehabilitation - Cerebral vascular accident - Cerebrovascular accident - Cerebral vascular incident - Cerebrovascular incident - Vascular cerebral accident - Vascular cerebral incident - Cerebral incident	- Healthy patients - Musculoskeletal patients - Spinal cord injury - Other neurological patients/impairments - Spasticity due to other causes
Intervention	- Dry needling - Needling - DN - DNHS	- Acupuncture - Electric stimulation of dry needling - All other interventions
Outcome	- Muscle tonus - Muscular properties - Muscle architecture - Muscle morphology (Cross-sectional area, pennation angle, muscle thickness, fascicle length muscle length) - Elastography (Stiffness, elastic stiffness, intrinsic stiffness) - Muscle impedance - Myoton - Torque - Range of motion, articular - Passive range of motion - ROM - Neurophysiologic changes (EMG, Hoffmann reflex, reflex torque, catch angle)	- Active torque - Active range of motion - Other non-related outcomes

**Table 2:** Final search strategy (2 February 2026).

Table Heading	Search Strategy	Results
PubMed	(((“Stroke”[MeSH Terms]) OR (“stroke rehabilitation”) OR (“cerebrovascular accident”) OR (CVA) OR (“cerebral incident”)) AND ((“Muscle Spasticity”[MeSH Terms]) OR (spasticity)) AND ((“Dry Needling”[MeSH Terms]) OR (DN) OR (DNHS) OR (“dry needl*”) OR (“needling”) OR (“acupuncture needle”) NOT (“Acupuncture”[MeSH Terms])) AND (((“Muscle Tonus”[MeSH Terms]) OR (“muscular properties”) OR (“muscular architecture”) OR (“muscle architecture”) OR (“muscle morphology”) OR (elastography) OR (“muscle impedance”) OR (“cross sectional area”) OR (“pennation angle”) OR (“muscle thickness”) OR (“muscle length”) OR (stiffness) OR (viscosity) OR (visco-elastic) OR (myoton) OR (torque) OR (“range of motion, articular”[MeSH Terms]) OR (“passive range of motion”) OR (ROM) OR (“passive ROM”) OR (PROM)) OR (electromyograph* OR EMG OR reflex* OR Tardieu OR TMS OR “transcranial magnetic stimulation”)))	35
Web of Science	(ALL = ((stroke) OR (stroke rehabilitation) OR (cerebrovascular accident) OR (CVA) OR (cerebral incident))) AND (ALL = ((spasticity) OR (muscle spasticity))) AND (ALL = ((dry needling) OR (DN) OR (DNHS) OR (dry needl*) OR (needling) OR (acupuncture needle)) NOT ALL = (acupuncture)) AND (ALL = ((muscle tonus) OR (muscular properties) OR (muscle architecture)OR (muscle morphology) OR (elastography) OR (muscle impedance)OR (cross sectional area) OR (pennation angle) OR (muscle thickness)OR (muscle length) OR (stiffness) OR (viscosity) OR (visco-elasticity) OR (myoton) OR (torque) OR (range of motion) OR (passive range of motion) OR (ROM) OR (passive ROM) OR (PROM)) OR ALL = (electromyograph* OR EMG OR reflex* OR Tardieu OR TMS OR “transcranial magnetic stimulation”))	81
Scopus	(TITLE-ABS-KEY ((stroke OR “cerebrovascular accident” OR CVA OR “cerebral incident” OR (stroke AND rehabilitation)) AND (spasticity OR “muscle spasticity”) AND (“dry needling” OR DN OR DNHS OR needling OR (acupuncture AND needling))) AND NOT TITLE-ABS-KEY (acupuncture) AND TITLE-ABS-KEY (“muscle tonus” OR “muscular properties” OR “muscular architecture” OR “muscle architecture” OR “muscle morphology” OR elastography OR “muscle impedance” OR “cross sectional area” OR “pennation angle” OR “muscle thickness” OR “muscle length” OR stiffness OR viscosity OR “visco-elastic” OR myoton OR torque OR “range of motion” OR “passive range of motion” OR ROM OR PROM OR “passive ROM”) OR ALL (electromyograph OR EMG OR reflex OR Tardieu OR TMS OR “transcranial magnetic stimulation”))	62
Embase	(‘stroke’/exp OR stroke:ab,ti OR ‘cerebrovascular accident’:ab,ti OR cva:ab,ti OR ‘cerebral incident’:ab,ti) AND (‘muscle spasticity’/exp OR spasticity:ab,ti OR ‘muscle spasticity’:ab,ti) AND (‘dry needling’/exp OR ‘dry needl*’:ab,ti OR dn:ab,ti OR dnhs:ab,ti OR ‘acupuncture needling’:ab,ti) NOT (‘acupuncture’/exp OR acupuncture:ab,ti) AND (‘muscle tonus’:ab,ti OR ‘muscular properties’:ab,ti OR ‘muscular architecture’:ab,ti OR ‘muscle architecture’:ab,ti OR ‘muscle morphology’:ab,ti OR ‘elastography’/exp OR elastography:ab,ti OR ‘muscle impedance’:ab,ti OR ‘cross sectional area’:ab,ti OR ‘pennation angle’:ab,ti OR ‘muscle thickness’:ab,ti OR ‘muscle length’:ab,ti OR stiffness:ab,ti OR ‘muscle stiffness’/exp OR viscosity:ab,ti OR ‘viscoelasticity’/exp OR ‘visco elastic’:ab,ti OR myoton:ab,ti OR torque:ab,ti OR ‘range of motion’:ab,ti OR ‘passive range of motion’:ab,ti OR rom:ab,ti OR prom:ab,ti OR electromyograph*:ab,ti OR emg:ab,ti OR reflex*:ab,ti OR tardieu:ab,ti OR tms:ab,ti OR ‘transcranial magnetic stimulation’:ab,ti)	48

## Data Availability

Other datasets generated and/or analyzed during the current study are available from the corresponding author on reasonable request.

## Supplementary Materials

The following supporting information can be downloaded at: https://www.mdpi.com/article/10.3390/jcm15114246/s1, PRISMA 2020 Checklist for systematic reviews.
